# The prognostic impact of epidermal growth factor receptor in patients with metastatic gastric cancer

**DOI:** 10.1186/1471-2407-12-524

**Published:** 2012-11-15

**Authors:** Akin Atmaca, Dominique Werner, Claudia Pauligk, Kristina Steinmetz, Ralph Wirtz, Hans-Michael Altmannsberger, Elke Jäger, Salah-Eddin Al-Batran

**Affiliations:** 1 Department of Hematology and Oncology, Institute of clinical research (IKF) at Krankenhaus Nordwest, UCT-University Cancer Center, Frankfurt am Main, Germany; 2 STRATIFYER Molecular Pathology GmbH, Köln, Germany; 3 Institute for Pathology, Krankenhaus Nordwest, Frankfurt am Main, Germany

**Keywords:** EGFR, Immunohistochemistry, Gastric cancer, Survival, Prognostic

## Abstract

**Background:**

The epidermal growth factor receptor (EGFR) is a potential target of anticancer therapy in gastric cancer. However, its prognostic role in metastatic gastric or gastroesophageal junction (GE) cancer has not been established yet.

**Methods:**

EGFR status was analyzed by immunohistochemistry (IHC) in paraffin-embedded samples from 357 patients who received chemotherapy in 4 first-line trials. Automated RNA extraction from paraffin and RT-quantitative PCR were additionally used to evaluate EGFR mRNA expression in 130 patients.

**Results:**

EGFR protein expression (any grade) and overexpression (3+) were observed in 43% and 11% of patients, respectively. EGFR positivity correlated with intestinal type histology (*p* = 0.05), but not with other clinicopathologic characteristics. Median follow-up was 18.2 months. Median overall survival (OS) was similar in patients with EGFR positive vs. those with EGFR negative tumors, regardless whether positivity was defined as ≥1+ (10.6 vs. 10.9 months, *p* = 0.463) or as 3+ (8.6 vs. 10.8 months, *p* = 0.377). The multivariate analysis indicated that EGFR status is not an independent prognostic factor (hazard ratio 0.85, 0.56 to 1.12, *p* = 0.247). There were also no significant differences in overall survival when patients were categorized according to median (*p* = 0.116) or quartile (*p* = 0.767) distribution of EGFR mRNA gene expression. Similar distributions of progression-free survival according to EGFR status were observed.

**Conclusions:**

Unlike different cancer types where EGFR-positive disease is associated with an adverse prognostic value, EGFR positivity is not prognostic of patient outcome in metastatic gastric or GE cancer.

## Background

Despite reasonable improvement in the therapeutic management of advanced gastric cancer with new active regimens [1-3], the prognosis is still very limited, with a median overall survival of approximately 9 to 11 months. With the emergence of new therapeutic options, great effort is made in the research of biomarkers, which can help to identify subgroups of patients, who may benefit from special treatments. To date, human epidermal growth factor receptor 2 (HER2) overexpression (observed in up to 22% of patients) is the only predictive factor, which predicts a benefit from a treatment with the anti-HER2 antibody trastuzumab [4].

Aberrant epidermal growth factor receptor (EGFR) signaling plays an important role in development and progression of various human tumors. EGFR has been demonstrated to phosphorylate and regulate numerous cellular proteins and to initiate several signal transduction cascades, which lead to cell proliferation, migration, invasion, metastasis, angiogenesis and inhibition of apoptosis. While EGFR inhibitors for metastatic gastric cancer are currently under investigation, the prognostic role of EGFR in gastric cancer remains controversial.

While many initial reports indicated unfavorable outcome for EGFR protein expression or overexpression in patients with resectable gastric cancer [5-11] some recent reports could not observe a correlation between EGFR expression and survival [12], or even found a significant correlation of high EGFR expression with favorable outcome in patients with curatively resected gastric cancer [13].

Regarding these particular controversial findings, which are partly based on different patient populations and different analysis methods, there is a need for clarifying the role of EGFR expression in a distinct setting and well defined patient population.

To clarify the clinical relevance of EGFR status, this study examines the clinicopathologic characteristics and outcomes in a uniform population of Western patients with gastric/GE junction adenocarcinoma enrolled in first-line metastatic chemotherapy trials.

## Methods

### Patients

Stage IV gastric cancer patients with available tissue for EGFR testing were indentified from a prospective database of four first-line trials of chemotherapy [14-17].

Patients gave written informed consent on participation in the clinical trial and on sample collection and analysis, which was approved by the responsible ethic committee (ethics committee of the Landesärztekammer Hessen, Germany). Standards of the International Conference on Harmonization/ World Health Organization (WHO) Good Clinical Practice were followed.

### Pathology review

Formalin-fixed paraffin-embedded (FFPE) tumor samples were evaluated for EGFR protein expression by immunohistochemistry (IHC). For each case, a corresponding hematoxylin–eosin (H&E) section was reviewed to assess the sample adequacy. All tumors were re-examined by a gastrointestinal pathologist (HA) to confirm the histological subtype (diffuse vs. intestinal vs. mixed).

### Immunohistochemistry

Tissue sections (5 μm thickness) were stained with H&E or immunostained by indirect immunoperoxidase method (DAKO, Glostrup, Denmark) as recommended by the manufacturer. For detection of EGFR the EGFR pharmDx TM Kit for the Dako Autostainer was used. Tissue staining was visualized with a DAB substrate chromogen solution. Slides were counterstained with hematoxilin, dehydrated and mounted.

Membrane staining was evaluated in the neoplastic cells and quantified and graded as recommended in the detection kit (primary scoring system): 0, no staining or membranous reactivity in <10% of tumor cells; 1+, weak, barely perceptible membranous reactivity in > 10% of tumor cells; 2+, complete or basolateral membranous reactivity either non-uniform or weak in at least 10% of cells; 3+, complete or basolateral membranous reactivity of strong intensity in ≥10% of cells. Additionally the following two scoring systems were also evaluated:

1.) H-Score: The score was obtained by the formula: 3 x percentage of strongly staining nuclei + 2 x percentage of moderately staining nuclei + percentage of weakly staining nuclei, giving a range of 0 to 300. Samples with score >200 were classified as positive (overexpression) [18].

2.) Modified semiquantitative H-score: intensity of staining from 0 to 3 multiplied by the percentage of positive tumor cells, which were categorized as 0.1 for 1-9%, 0.5 for 10-49% and 1.0 for >50% positive tumor cells. A score >1.0 was classified as positive [19].

### RNA extraction and gene expression analysis

Formalin-fixed paraffin-embedded (FFPE) tumor samples were evaluated for mRNA expression. From each tumor block, a 5-μm section was stained with hematoxylin–eosin and revised by a pathologist and two consecutive 10-μm sections were cut on a standard microtome, tumor was macro-dissected and placed into individual tubes, and stored at 4^0^C for ~1 month until RNA extraction. Fully automated high-throughput RNA extraction has been carried out according to methods previously published [20].

Expression of EGFR and the normalization (housekeeping) gene RPL37A was assessed by one-step RT-quantitative PCR (qPCR). SuperScript ® III Platinum ® One-Step qRT-PCR System with ROX (Invitrogen, Karlsruhe, Germany) was used according to the manufacturer’s instructions. Experiments were carried out on an ABI PRISM ®7900HT (Applied Biosystems, Darmstadt, Germany) with 30 min at 50°C, 2 min at 95°C followed by 40 cycles of 15 s at 95°C and 30 s at 60°C. Relative copy numbers positively correlating with the expression of the genes of interest were calculated by using the 2(40-DDCT)-method. Each mRNA expression was adjusted with the housekeeping gene. For assessment of DNA contamination in RNA preparations, a PAEP gene-specific qPCR without preceding reverse transcription was carried out using the reagents from the SuperScript III ® Platinum ® One-Step qRTPCR System with ROX and Taq DNA Polymerase. In samples with a Cq value <35, the DNase I treatments were repeated to prevent effects on bispecific PCR assays. Stratagene human QPCR Reference total RNA (Stratagene, Waldbronn, Germany) was used as positive control for RTqPCR and human genomic DNA (Roche Diagnostics, Basel, Switzerland) as positive control for qPCR. All PCR assays were carried out in triplicate, and the mean of triplicates was reported. Kinetic RT-PCR was applied for the assessment of mRNA expression using the following TaqMan™-based primer/probe set™-based primer/probe set (Eurogentec, Seraing, Belgium):

EGFR probe CCTTGCCGCAAAGTGTGTAACGGAAT.

Forward primer CGCAAGTGTAAGAAGTGCGAA.

Reverse primer CGTAGCATTTATGGAGAGTGAGTCT.

### Statistical analysis

Progression-free survival (PFS) and overall survival (OS) were calculated by the Kaplan–Meier method, and statistical significance was analyzed using the log-rank test. To assess the univariate relationship between clinicopathologic variables and EGFR-status (positive or negative), the Fishers’ exact test was applied. Furthermore, Cox proportional hazard models were used for the multivariate analyses concerning survival times. All *p* values were two-sided, with *p* values <0.05 considered to indicate statistical significance.

## Results

### Patient characteristics

The cohort consists of 357 patients with stage IV adenocarcinoma of middle to distal stomach (65%) or GE junction (30%), with a similar number of Lauren’s diffuse/mixed (48%) and intestinal tumors (39%). Liver was the most common site of metastatic disease (44%). The majority of samples were from primary tumor (83%) and were biopsy specimens (67%). Patients predominantly presented with metastatic disease (83%), and went on to receive three-drug combination chemotherapy (54%). Table 1 summarizes patient characteristics.

**Table 1:** Patient Characteristics (N = 357)

**Characteristic**	**Patients**
	**N (%)**
Age, median (range)	
Sex	
Male	214 (60)
Female	143 (40)
ECOG performance status 0-1	326 (91)
Primary tumor location	
Gastroesophageal junction/proximal stomach	107 (30)
Mid to distal stomach	231 (65)
Unclassifiable/Unknown	19 (5)
Disease status	
Stage IV at diagnosis	298 (83)
z Recurrent disease	59 (17)
First-line chemotherapy	
3-drug combination (FLOT)	192 (54)
2-drug combination (FLO or FLP)	165 (46)
Metastatic disease sites	
Liver	157 (44)
Lymph nodes	219 (61)
Peritoneum	93 (26)
Lung	60 (17)
Lauren classification	
Diffuse/mixed	170 (48)
Intestinal	139 (39)
Other/Unknown	48 (13)
Sampling specimen	
Biopsy	238 (67)
Surgical specimen	116 (33)
Unknown	3 (1)
Primary tumor	298 (83)
Metastatic lesion	50 (14)
Unknown	9 (3)

Abbreviations: FLOT, 5-FU, leucovorin, oxaliplatin and docetaxel; FLO, 5-FU, leucovorin, oxaliplatin; FLP, 5-FU, folic acid, cisplatin.

5-FU, Leucovorin and Oxaliplatin (FLO), 5-FU, Folic acid and Cisplatin (FLP).

The proportion of patients randomized in four different first-line trials with available tumor samples was 67.7% [14], 58.3% [15], 28.7% [16], and 52.4% [17], respectively. There was no significant difference in patients’ characteristics, regarding patients with available tumor samples and the entire study cohort.

One hundred ninety-two patients (54%) received a three-drug regimen of oxaliplatin 85 mg/m^2^, leucovorin 200 mg/m^2^, and fluorouracil 2600 mg/m^2^ as a 24-hour infusion in combination with docetaxel 50 mg/m^2^ (FLOT) on day 1 every 2 weeks [15-17]. One hundred sixty-five patients (46%) received a regimen of fluorouracil 2,600 mg/m^2^ via 24-hour infusion, leucovorin 200 mg/m^2^, and oxaliplatin 85 mg/m^2^ (FLO) every 2 weeks, or fluorouracil 2,000 mg/m^2^ via 24-hour infusion, leucovorin 200 mg/m^2^ weekly, and cisplatin 50 mg/m^2^ every 2 weeks (FLP) [14].

### EGFR protein expression and correlation to clinicopathologic characteristics

On IHC and according to the primary scoring system, 152 of 357 (43 %) patients tested EGFR-positive. EGFR was negative, 1+, 2+, and 3+ in 205 (57%), 50 (14%), 62 (17%), and 40 (11%) patients, respectively. With the modified semiquantitative H-score, 116 (33%) patients were classified as EGFR positive (>score 1.0) and 30 (8%) patients had an H-score of >200 (EGFR overexpression).

Table 2 summarizes EGFR positivity rate according to baseline characteristics. The rate of EGFR positivity (any grade) was similar between biopsies and surgical specimens (41% vs 47% *p* = 0.538), primary tumor and metastasis (42% vs 52% *p* = 0.418), histological subtypes (Lauren’s intestinal type 48% vs 37% in diffuse type, *p* = 0.211), in the proximal region encompassing gastric cardia/GE junction location and in the gastric body and antrum (44% vs. 43%, *p* = 0.916) and finally according to metastatic site (e.g. liver metastasis or not, 46% vs. 40%, *p* = 0.559).

**Table 2:** EGFR Positivity by Study Subgroup

**Characteristic, *n *= 357**	**EGFR neg**	**EGFR pos**	***P***
	**(score 0)**	**(score 1-3+)**	**Value**
	**n (%)**	**n (%)**	
Female, 143	79 (55)	64 (45)	
Male, 214	126 (59)	88 (41)	.69
Age,			
>65, 173	96 (55)	77 (45)	
≤65, 181	107 (59)	74 (41)	.698
ECOG performance status,			
0-1, 326	186 (57)	140 (43)	
2, 22	11 (50)	11 (50)	.697
Not specified, 9	8 (89)	1 (11)	
Primary tumor location			
Gastroesophageal junction, 107	60 (56)	47 (44)	
Mid to distal stomach, 231	132 (57)	99 (43)	.916
Not specified, 19	13 (68)	6 (32)	
Disease status			
Stage IV at diagnosis, 298	171 (57)	127 (43)	
Recurrent disease, 59	34 (58)	25 (42)	1
Metastatic disease sites			
Liver, present, 157	85 (54)	72 (46)	
Liver, not present, 199	119 (60)	80 (40)	.559
Lymph nodes, present 219	127 (58)	92 (42)	
Lymph nodes, not present, 98	51 (52)	47 (48)	.585
Peritoneum, present ,93	57 (61)	36 (39)	
Peritoneum, not present, 263	147 (56)	116 (44)	.58
Lauren classification,			
Diffuse/mixed, 170	107 (63)	63 (37)	.211
Intestinal, 139	72 (52)	67 (48)	
Not specified, 48	26 (54)	22 (46)	
Specimen used for EGFR testing			
Biopsy, 238	141 (59)	97 (41)	
Surgical specimen,116	62 (53)	54 (47)	.538
Not specified, 3	2 (67)	1 (33)	
Primary tumor, 298	173 (58)	125 (42)	
Metastatic lesion, 50	24 (48)	26 (52)	.418
Not specified, 9	9 (100)	0	

Similar associations with clinicopathologic criteria were obtained when EGFR overexpression (EGFR 3+ group) was considered as positive, with the exception of histological subtype. Intestinal type tumors showed significantly higher rates of EGFR 3+ expression compared with diffuse/mixed type histology (16% vs. 8%, *p* = 0.05).

### Correlation between EGFR protein expression and survival

**Figure 1:**
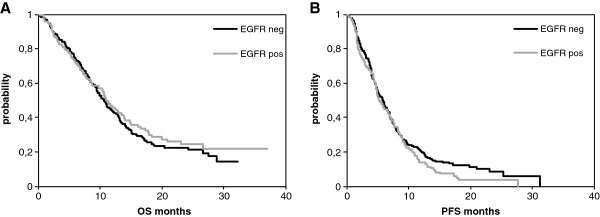
**Kaplan-Meier curves for (A) overall survival (OS) and (B) progression free survival (PFS) for patients with EGFR-positive (*n *= 152) and EGFR-negative (*n *= 205) disease.** Median OS: 10.9 vs 10.6 months, *p* = 0.463; median PFS: 5.3 vs 5.7 months, *p* = 0.185.

A total of 297 deaths (83%) had occurred at the time of analysis, with median follow-up for surviving patients of 18.2 months (range 3.3 to 44.1).There was no difference in OS and PFS between patients with EGFR positive and negative tumors with median OS and PFS being 10.9 vs.10.6 months (*p* = 0.463) and 5.3 vs 5.7 months (*p* = 0.185), respectively (Figure 1). There was either no statistical difference regarding OS and PFS between the different EGFR + subgroups (1+ to 3+; P for trend = 0.581).

Median OS and PFS showed also no statistical difference when the modified semiquantitative H-score (>1.0 vs ≤1.0) was applied (*p* = 0.544 and *p* = 0.325, respectively; data not shown). For the group with high EGFR-expression classified by the quantiative H- score (>200), OS and PFS also remained not statistically different from those found in the lower expression group (*p* = 0.764 and *p* = 0.272, respectively; data not shown).

Similar distributions of OS according to EGFR status were also observed when the survival analysis was adjusted for the use of docetaxel (yes or no; *p* = 0.390) and the type of platinum used (oxaliplatin or cisplatin; *p* = 0.337).

In the multivariate analysis, including EGFR status, age, sex, two- vs. three-drug chemotherapy, histologic subtype, and disease status, EGFR status was not an independent predictor of overall survival (HR 0.85; *p* = 0.247; Table 3). In the multivariate analysis only three-drug chemotherapy was significantly associated with longer survival time.

**Table 3:** Univariate and multivariate overall survival analyses

**Characteristic**	**Hazard ratio (95% CI)**	***P- value***
EGFR positive vs EGFR negative	0.91 (0.66 to 1.16)	.464
Age >65 vs ≤65	1.04 (0.80 to 1.29)	.747
ECOG performance status 2–3 *vs* 0-1	2.13 (1.66 to 2.60)	**.002**
Therapy with 2- drug vs 3- drug combination	0.82 (0.58 to 1.07)	.117
gastroesophageal junction vs Stomach	0.69 (0.40 to 0.97)	**.009**
recurrent disease vs Stage IV at diagnosis	0.84 (0.50 to 1.18)	.326
Male *vs* female	0.92 (0.67 to 1.17)	.512
Liver metastasis, yes vs no	1.19 (0.95 to 1.44)	.163
Peritoneal metastasis, yes vs no	1.26 (1.00 to 1.52)	.079
Intestinal vs diffuse/mixed	0.90 (0.63 to 1.16)	.43
Multivariate Overall Survival Analysis		
Characteristic	Hazard ratio (95% CI)	*P- value*
EGFR positive vs EGFR negative	0.85 (0.57 to 1.13)	.247
Age >65 vs ≤65	1.16 (0.88 to 1.45)	.299
Therapy with 2- drug vs 3- drug combination	0.71 (0.39 to 0.97)	**.033**
gastroesophageal junction vs Stomach	0.78 (0.43 to 1.14)	.176
recurrent disease vs Stage IV at diagnosis	0.71 (0.33 to 1.10)	.087
Male *vs* female	1.01 (0.71 to 1.30)	.959
Intestinal vs. diffuse/mixed	0.9 (0.59 to 1.22)	.517

^a^ Univariate Overall Survival Analysis.

### Correlation between EGFR mRNA gene expression and survival

**Figure 2:**
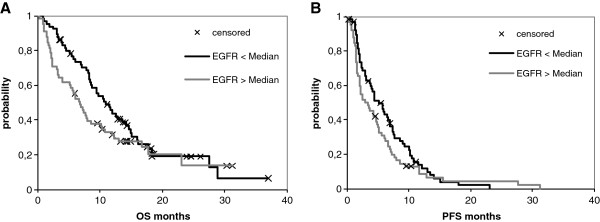
**Kaplan-Meier curves for (A) overall survival (OS) and (B) progression free survival (PFS) for patients with EGFR-mRNA > median expression (*n *= 65) and EGFR-mRNA < median expression (*n *= 65).** Median OS: 6.8 vs 10.7 months, *p* = 0.173; median PFS: 2.9 vs 5.9 months, *p* = 0.116.

In addition to EGFR IHC, EGFR mRNA expression was analyzed by realtime PCR in 130 of the 357 patients. EGFR mRNA levels correlated with protein levels in the tumor tissue. The median mRNA expression in patients with no EGFR protein expression was 252 copies versus a median of 298 copies in EGFR positive patients. The trend was also clearly visible in the different intensity grades of EGFR IHC staining (EGFR 1+: median mRNA 288; EGFR 2+: median mRNA 264; EGFR 3+: median mRNA 410). In line with the IHC results for protein expression, EGFR mRNA expression levels showed no significant correlation to overall or progression free survival. This was observed when the median was chosen as threshold (Figure 2) or when patients were grouped according to their quartile expression of EGFR mRNA (data not shown).

## Discussion

Our results show that EGFR status is not prognostic of patient outcome in metastatic gastric and GE junction adenocarcinoma. We also found no impact of EGFR status on progression-free survival, indicating that EGFR overexpression is not associated with more aggressive tumor biology or with resistance to chemotherapy in gastric and GE junction adenocarcinoma. Our analysis is based on a large and uniform cohort of Western patients with metastatic gastric cancer, all treated with standardized chemotherapy in a clinical trial. EGFR testing was performed according to different scoring systems and methods (protein and mRNA gene expression) reviewed by referenced pathologists, with other clinical and pathological characteristics captured prospectively in research databases. Unlike other studies, our cohort consists solely of patients with stage IV disease with well annotated chemotherapy data available on all of our patients, and none received EGFR targeted therapies in the first- or second-line setting. Our cohort is by far the largest (*n* = 357) reporting on the prognostic effect of EGFR on metastatic disease gastric cancer. Previous data from patients with metastatic gastric cancer are limited to two cohorts of 86 and 43 patients and have delivered conflicted results regarding the prognostic value [10,21].

Regarding the curable stages, more data exist but the prognostic role of EGFR expression in operable gastric cancer remains controversial. Expression of EGFR in resected gastric cancer has been linked to shorter overall survival, more advanced tumor stage, and lymph node metastases in some studies, but not in others [5-9,12,13]. For example, Kim et al. [13] found a correlation of EGFR expression and improved overall survival in patients with resected gastric cancer receiving adjuvant chemotherapy.

The controversial findings in resectable stages may particularly derive from missing standardized procedures and the lack of an established scoring system in the immunohistochemical evaluation of EGFR. Besides proper definition of the target population and tumor characteristics, it is important to reflect distinct information of immunohistochemical EGFR expression like intensity of staining, staining pattern (focal or homogenous), content of tumor cells and choice of primary antibodies.

Although EGFR is not a prognostic factor in metastatic gastric cancer, this is not a reflection on its value as a predictive marker. This is in line with recent results from HER2, which is an established predictive factor for treatment response to trastuzumab, while (according to recent reports) Her2 expression itself is not a prognostic factor in metastatic gastric cancer [4,22,23].

While no associations between Her2 expression (any grade) and clinicopathologic criteria were seen, we found that the rate of EGFR 3+ status was significantly associated with intestinal type histology (intestinal, 16%; diffuse, 8%; *p* = 0.05). The same pattern is known form Her2 expression [4,22] and may indicate a link between high expression of the erb-receptor family and a distinct disease biology in gastric cancer. In the TOGA-trial [4] it was shown that the extent of Her2 expression is of relevant predictive value. It was clearly demonstrated, that anti HER2 treatment is only reasonable in patients with high intensity HER2 expression (HER2 score 3+). Whether this observation will be applicable to EGFR inhibitors is unclear.

With emerging development of drugs interacting with the EGF receptor or the EGFR pathway with monoclonal antibodies like cetuximab and panitumumab, there is an enormous need of better understanding the way of interaction of these drugs and the need of identifying subgroups of patients, who are likely to have a clinical benefit.

In the clinical setting, anti EGFR antibodies seem to enhance the activity of chemotherapy with improved response rates up to 60% [24]. But recently presented results of a phase III study (REAL-3, [25]) comparing a first-line palliative chemotherapy (epirubicin, oxaliplatin and capecitabin) with or without the anti-EGFR antibody panitumumab, could not demonstrate an OS/PFS benefit or even showed a worse outcome in patients in the experimental arm.

It will be interesting if these studies could identify molecular subgroups of patients, who nevertheless could benefit from an EGFR targeted treatment.

## Conclusion

Unlike different cancer types where EGFR-positive disease is associated with an adverse prognostic value, EGFR positivity is not prognostic of patient outcome in metastatic gastric or GE cancer.

## Competing interest

The authors declare that there are no conflicts of interest.

## Authors’ contribution

AA carried out the analysis of the immunohistochemistry and drafted the manuscript, DW carried out the immunohistochemistry and the statistical analysis, CP participated in the design of the study and performed the statistical analysis, KS carried out the immunohistochemistry, RW carried out the mRNA analysis, HA carried out the analysis of the immunohistochemistry, JE participated in the design and coordination of the study, SA designed and coordinated the study, carried out the immunhistochemistry and helped to draft the manuscript. All authors read and approved the final manuscript.

## Pre-publication history

The pre-publication history for this paper can be accessed here:

http://www.biomedcentral.com/1471-2407/12/524/prepub
